# Neonatal intensive care unit (NICU) exposures exert a sustained influence on the progression of gut microbiota and metabolome in the first year of life

**DOI:** 10.1038/s41598-020-80278-1

**Published:** 2021-01-14

**Authors:** Polly Soo Xi Yap, Chun Wie Chong, Azanna Ahmad Kamar, Ivan Kok Seng Yap, Yao Mun Choo, Nai Ming Lai, Cindy Shuan Ju Teh

**Affiliations:** 1 Department of Medical Microbiology, Faculty of Medicine, University of Malaya, 50603 Kuala Lumpur, Malaysia; 2 School of Pharmacy, Monash University Malaysia, 47500 Bandar Sunway, Selangor Malaysia; 3 Neonatal Intensive Care Unit (NICU), Department of Paediatrics, Faculty of Medicine, University of Malaya, 50603 Kuala Lumpur, Malaysia; 4 Sarawak Research and Development Council, 11th Floor LCDA Tower, The Isthmus, 93050 Kuching, Sarawak Malaysia; 5 School of Medicine, Faculty of Health and Medical Sciences, Taylor’s University, 47500 Subang Jaya, Selangor Malaysia

**Keywords:** Biotechnology, Computational biology and bioinformatics, Microbiology, Medical research, Molecular medicine

## Abstract

Emerging evidence has shown a link between the perturbations and development of the gut microbiota in infants with their immediate and long-term health. To better understand the assembly of the gut microbiota in preterm infants, faecal samples were longitudinally collected from the preterm (n = 19) and term (n = 20) infants from birth until month 12. 16S rRNA gene sequencing (n = 141) and metabolomics profiling (n = 141) using nuclear magnetic resonance spectroscopy identified significant differences between groups in various time points. A panel of amino acid metabolites and central metabolism intermediates significantly correlated with the relative abundances of 8 species of bacteria were identified in the preterm group. In contrast, faecal metabolites of term infants had significantly higher levels of metabolites which are commonly found in milk such as fucose and β-hydroxybutyrate. We demonstrated that the early-life factors such as gestational age, birth weight and NICU exposures, exerted a sustained effect to the dynamics of gut microbial composition and metabolism of the neonates up to one year of age. Thus, our findings suggest that intervention at this early time could provide ‘metabolic rescue’ to preterm infants from aberrant initial gut microbial colonisation and succession.

## Introduction

Preterm birth is one of the major determinants of neonatal mortality and morbidity. Prematurity is also well-recognised to carry long term consequences on health well into the infants’ adult life. Children born prematurely have higher rates of developing diabetes^1,2^, hypertensions^3,4^ and obesity^5^ as compared to those born at term. Large cohort study on the longitudinal maturation of full term infant gut microbiome showed three distinct phases of microbiome progression at first year, between second to third year and beyond three years of life^6^. Further, the rate of gut microbiome maturation accelerated after the cessation of breast-feeding^6,7^. Additionally, there have been various studies of patterns in gut microbiome of preterm infants in the last two decades, most reporting correlations between the gut microbiota and various factors during early life, such as gestational age^8,9^, breastmilk^10–12^ and antibiotic exposures^11,13^. Also, a number of studies have characterised the gut microbiome composition in infants subjected to different modes of delivery^14^, modes of feeding^9^, and geographical locations^15,16^. However, limited studies have advanced interpretations on the impact of degree of prematurity at birth and postnatal environment (for e.g. treatments received in NICU) in terms of longitudinal associated patterns, especially gut microbiota maturation after weaning, despite increasing evidence of the population dynamics of the human gut microbiome^17^. Tauchi et al.^18^ studied the longitudinal stool samples of preterm neonates from NICU and observed an age-dependent transition from Gram-positive cocci-dominated microbiota to *Enterobacteriaceae* and/or *Bifidobacteriaceae*-dominated microbiota. Understanding how host factors influence diversity is an important step towards unravelling the relationship between gut microbial composition and the host health conditions. The integration of metagenomes and metabolomes provided a systemic view on how gut microbial ecosystems operate. Given that host metabolism is strongly influenced by gut microbes through their collective activities and cooperative responses, the association of health status and gut microbial dynamics should therefore be viewed holistically.

Based on the National Obstetrics Registry report in 2015, preterm birth rate in Malaysia was recorded at 12.4%^19^. Specifically, preterm birth rates were highest among the indigenous population in Peninsular Malaysia, mothers at extreme ages, parity > 6, and mothers with preeclampsia, eclampsia and renal disease^19^. The complications of preterm birth and morbidity arise from immature organ systems that are incapable of supporting life in the extra-uterine environment. Despite the significant health burden of preterm birth, the consequences of impaired maturation of gut microbiota to the development of preterm infants to adult life remained poorly studied, especially in South East Asia. Further, there is a lack of information regarding the contribution of gut microbial composition to host metabolism, and the interplay between both in the growth of preterm infants. There is therefore a need to characterise the temporal dynamics of the human gut microbiome and metabolome to understand their roles in the preterm infants’ health and development. In this study, we compared the variations in the faecal microbial composition and metabolome between preterm and term infants at varying time points from birth to month 12. The pattern of gut bacterial assemblages and metabolic profiles were modelled using selected clinical characteristics while the relationship of microbial taxa and metabolites that differed significantly across different time points was assessed through sparse partial least squares correlation analysis.

## Results

### Characteristics of the preterm and term infants

Of the 19 preterm infants studied, two were lost-to-follow-up due to early death. For comparison, a total of 20 healthy full-term infants born at a gestational age of more than 37 weeks were included in this study. Characteristics of the preterm and term infants are reported in Table 1. Term infants were mainly born by spontaneous vaginal delivery and no exposure to antibiotics while preterm infants were mainly born by caesarean delivery (94.7%, *P* < 0.0001) and exposed to antibiotics (57.9%, *P* < 0.0001). Clinical characteristics of the preterm infants during admission are reported in Table 2. Preterm infants’ stool samples collected during NICU admission were also subjected for bacterial isolation for multidrug resistant *Enterobacteriaceae*, as published in Yap et al.^20^. Antibiotic susceptibility profile of the isolates was correlated with patient clinical data for further statistical analyses.

**Table 1:** Demographics and characteristics of all infants studied.

	Preterm (*n* = 19 )	Term (*n* = 20 )	*P* value
Number of stool samples for omics studies	58	83	–
Mean gestational age, weeks (SD)	31.79 (2.97)	39.85 (0.81)	< 0.0001
Mean birth weight, g (SD)	1349.21 (515.98)	3036.5 (214.44)	< 0.0001
Delivery: caesarean/ vaginal	18/1	5/15	< 0.0001
Gender: male/female	8/11	10/10	0.6211
Race: Malay/Chinese/Indian	11/7/1	5/11/4	0.0865
Antibiotic treatment: yes/no	11/8	0/20	< 0.0001

*P* values < 0.05 were considered as significant.

*t* test for continuous data; Chi-square test for categorical data.

**Table 2:** Clinical characteristics of the preterm infants studied.

Infant	Gestational age (weeks)	Birth weight (g)	NICU stay (days)	Outcome at discharge	Parenteral nutrition (days)	Invasive ventilation (days)	Respiratory Distress Syndrome (RDS)	Antibiotherapy (days)	Sample collection*	
									Bacteria isolation^a^	16 s rRNA sequencing
B1	33	1570	21	Alive	0	0	No	0	m, w1, w2	m, m6, m12
B2	33	1725	13	Alive	0	0	No	0	m, w1	w1, m6, m12
B4	30	1510	44	Alive	20	0	Yes	12	m, w1, w2	m, w1, m6, m12
B6	35	1390	36	Alive	12	3	Yes	7	m, w1, w2	m, w1, m6, m12
B7	33	490	34	Dead	34	14	Yes	14	m, w1, w2	m, w1, w2
B8	30	1060	23	Alive	13	5	Yes	3	m	m, w1, w2
B9	36	1480	20	Alive	0	0	No	0	m, w1, w2	w1, w2, m6
B11	36	1555	29	Alive	0	0	No	7	m, w1, w2	m, w1, w2
B12	34	2845	6	Alive	0	0	No	0	m	m, m6
B13	32	1640	25	Alive	11	0	Yes	0	m, w1, w2	m, w1, w2, m6, m12
B14	32	785	57	Alive	10	3	Yes	4	m, w1, w2	m, w1, w2, m6, m12
B15	32	1540	25	Alive	0	0	Yes	3	m	m, w1, w2
B16	30	925	56	Alive	17	0	Yes	3	m, w2	m, w2
B17	30	1240	39	Alive	4	0	Yes	6	m, w2	w2
B18	30	1220	30	Alive	0	0	Yes	0	m, w2	m, w2
B22	23	550	18	Dead	8	18	Yes	10	m, w1	m, w1
B25	29	1125	42	Alive	14	0	No	9	m, w1, w2	m, w1, w2, m6
B26	33	1690	14	Alive	0	0	No	0	m, w1, w2	m, w1, m6, m12
B27	33	1295	33	Alive	6	0	No	0	m, w1, w2	m, w1
Median (95% CI)	32(31, 33)	1390 (1141, 1639)	25 (18, 32)		6 (2, 10)	0 (0, 2)		3 (1, 5)		

*m, meconium; w1, week 1; w2, week 2; m6, month 6; m12, month 12.

^a^Culture for samples collected during hospitalisation.

Eleven preterm infants had respiratory distress syndrome (RDS) and five patients received invasive mechanical ventilation. Eleven patients received parental nutrition (PN) and three of them had peripherally inserted central catheter (PICC). Eleven patients were treated with antibiotics for three days to two week with various combinations of penicillin (8 patients), gentamicin (10 patients), vancomycin (4 patients) and meropenem (4 patients).

Stool samples were collected at: day 1 (meconium), week 1, week 2, month 6 and month 12 of life from all infants. However, some time points were missed for selected infants due to the lack of adherence to the sampling schedule. In total, we collected 141 samples, for an average of 3.05 samples per preterm infants and 4.15 samples per term infants.

### Microbial composition comparison between term and preterm infants

**Figure 1:**
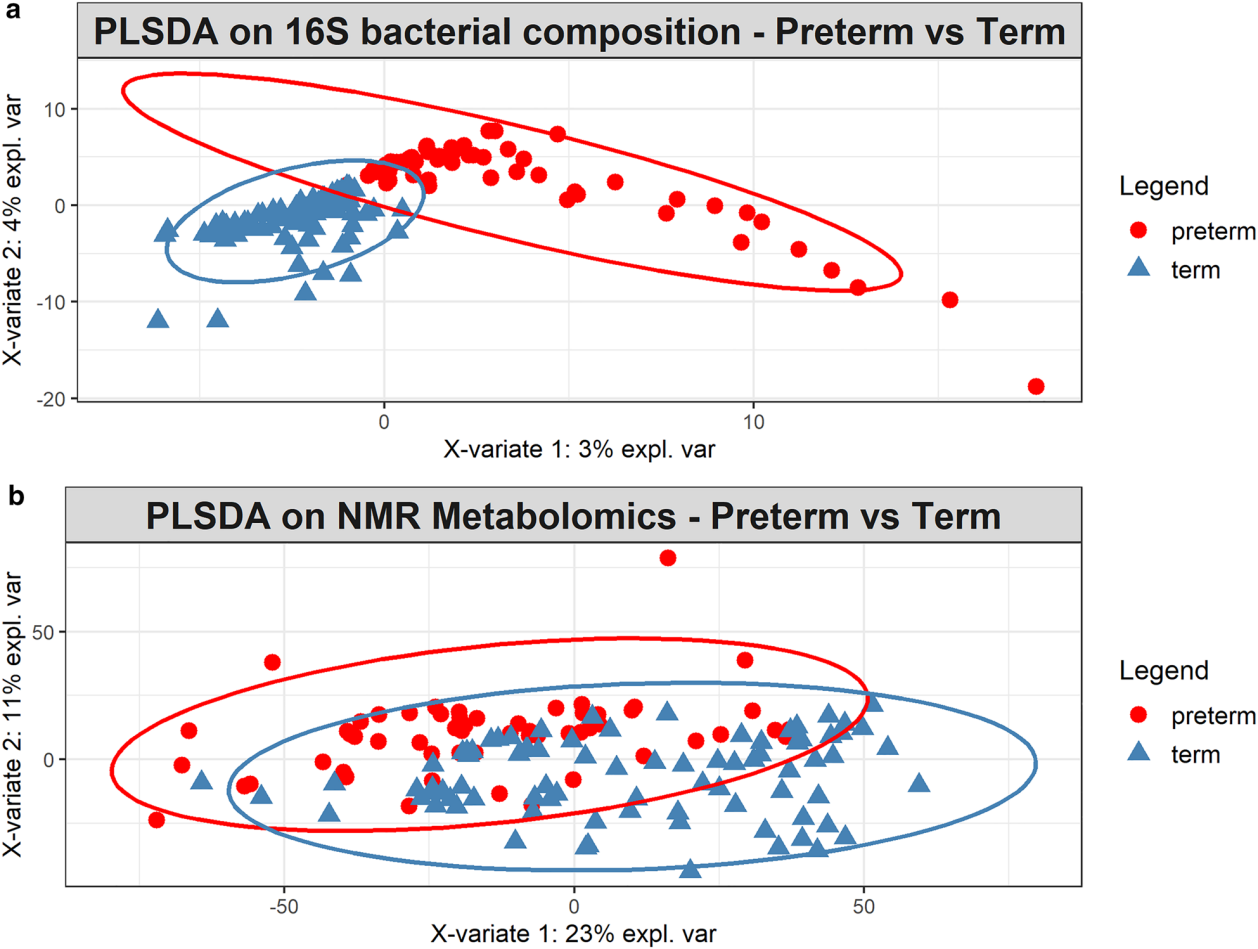
Faecal bacterial and metabolite composition of preterm and term control. PLS-DA models depicting inter-subject distances based on the (**a**) 16S bacterial composition (fivefold cross-validation, 2 components, overall error rate = 0.30) and (**b**) NMR metabolomics (fivefold cross-validation, 2 components, overall error rate = 0.29). Prevalence uncovers distinct clusters among the subject group. Eclipses for each group are calculated based on a 95% confidence level. PLS-DA plots were generated using R statistical software (version 3.5.0, https://cran.r-project.org/).

**Figure 2:**
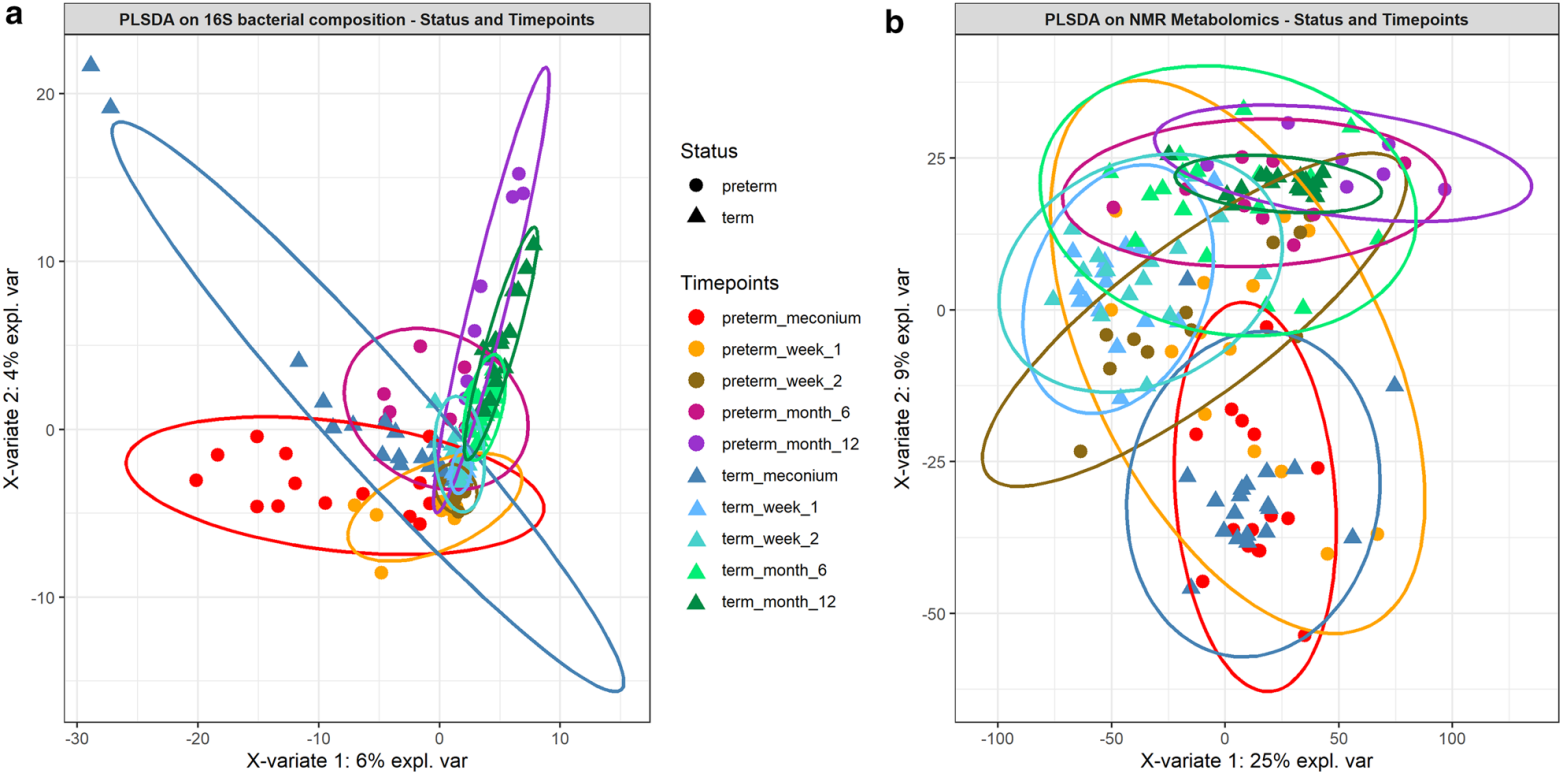
PLS-DA models of (**a**) 16S bacterial composition and (**b**) NMR metabolomics. The models were built on inter-subject vs the five collection time points and colour coded by group with 95% confidence eclipses. Both validated with fivefold cross-validation. PLS-DA plots were generated using R statistical software (version 3.5.0, https://cran.r-project.org/).

The alpha diversities (Shannon’s, Inverse Simpson’s, Pielou’s evenness, Chao1 and Faith’s phylogenetic diversity indices) of the faecal microbial community between groups (term and preterm) and between term and preterm after birth, week 1, week 2, month6 and month 12 were calculated. No significant difference in bacterial community richness, evenness and phylogenetic diversity were observed between term and preterm samples (Supplementary Fig. 1). Out of the five indices, only Faith's phylogenetic diversity showed significant difference (*P* < 0.01) between term and preterm at meconium and month 6 (Supplementary Fig. 2). Separately, beta diversity was assessed using PERMANOVA and PLS-DA. Overall, significant differences in faecal bacterial composition between the term and preterm infants were detected in PERMANOVA (pseudo-*F* = 2.4834, *P*(perm) = 0.001). The first PLS-DA plot (Fig. 1) showed clear separation in faecal bacterial compositions between term and preterm infants. When time points were added as factor, it was observed that samples from week 2 were overlapped with meconium and week 1 (Fig. 2). For month 6 and month 12, however, both groups formed two tight clusters with little overlapped. Consistent observation was also obtained using pairwise PERMANOVA analysis (Supplementary Table 2).

The overall distributions of the phyla and genera were provided in Supplementary Fig. 3. Differentially abundance OTUs were identified based on negative binomial model. A total of 55 OTUs from 3 phyla (Proteobacteria, Firmicutes and Bacteroidetes) whose abundance differed between two groups across the five time points were identified. The species which matched highest sequence homology with the input were included in the Supplementary Table 2. When the comparison was made at phylum level, the dominant phyla for term infants were Firmicutes and Bacteroidetes; while enriched levels of Proteobacteria were observed in preterm infants’ stool at the first two weeks of life. At 12 months of life, significant abundance of Bacteroidetes (OTU0012, OTU0029, OTU0051 and OTU0110) were observed among the preterm group. Conversely, term infants were observed with abundant species of Proteobacteria at month 12. The preterm infants’ stool samples had significantly enriched *Klebsiella* OTUs (mainly *K. pneumoniae*) during the first two weeks of life while the level of *Klebsiella* OTUs started to elevate in term infant stools only after 6 months of life. It was observed that OTU0029 (*Bacteroides fragilis*) was elevated in week 1 and week 2 stools of term infants while the significant elevation only observed at month 12 for preterm.

### Temporal differences in metabolomics profiles between term and preterm infants

Significant metabolites partitioning between both groups were established based on PLS-DA and PERMANOVA (*P*(perm) = 0.001, *P*(MC) = 0.003). PLS-DA plot inferred using metabolomics profiles showed less apparent separation in comparison to the microbial composition, although distinct clusters specific to term and preterm are still discernible (Fig. 1). When time points were added as factor, samples from meconium, week 1 and week 2 were loosely clustered especially among the preterm infants (Fig. 2). Consistent with bacterial composition, contrasting temporal response pertinent term vs preterm was also detected at month 6 (*P*(perm) = 0.005, *P*(MC) = 0.005). Nonetheless, both sample groups (i.e. term and preterm) from month 12 did not show significant difference with most of the data points overlapped with month 6. This observation was also reflected in the significant metabolites detected consistently in month 6 and month 12 in the term infants.

**Figure 3:**
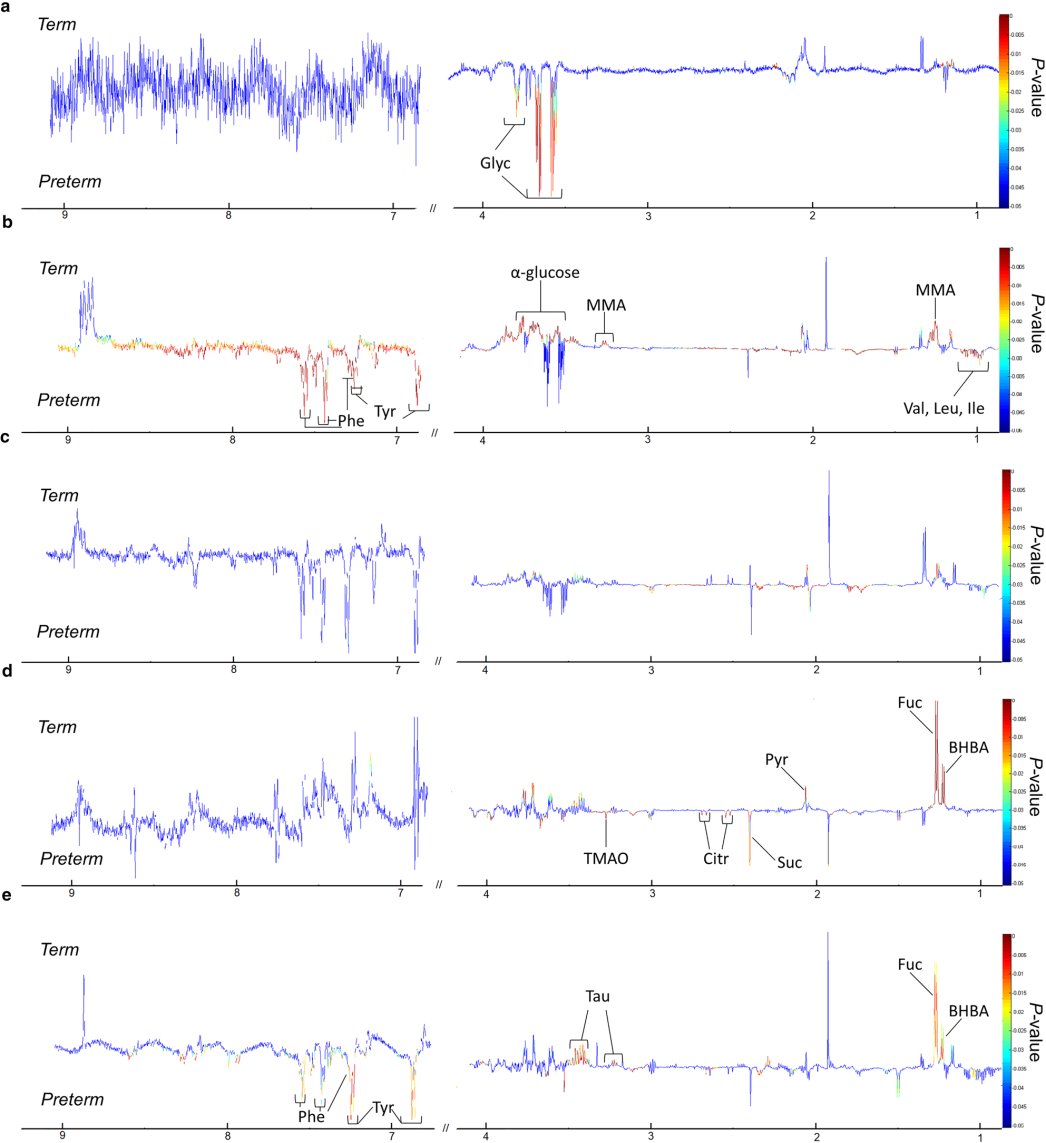
^1^H NMR spectroscopy showing the metabolite profiles of preterm and term controls. Covariance plots showing the colour-coded significance of stool metabolite profiles (red, *P* < 0.01) calculated using permutation test from (**a**) meconium, (**b**) week 1, (**c**) week 2, (**d**) month 6 and (**e**) month 12, were generated using MATLAB (version R2014a, https://www.mathworks.com) Key: BHBA, β-hydroxybutyrate; Citr, citrate; Fuc, fucose; Glyc, glycine; Ile, isoleucine; Leu, leucine; MMA, methylmalonic acid; Phe, phenylalanine; Pyr, pyruvatoxime; Suc, succinate; Tau, taurine; TMAO, trimethylamine-N-oxide; Tyr, tyrosine; Val, valine.

Statistical significance of the faecal metabolites was calculated using permutation test (number of permutations = 1000). Only metabolites with a *P* value of 0.01 and below detected in both groups were reported and summarised in Table 3. The corresponding covariance plots of preterm vs term derived from stools samples obtained from meconium, week 1, week 2, month 6 and month 12 of life were included in Fig. 3. Meconium collected from preterm infants showed elevated glycerol. At week 1, preterm group showed elevated faecal valine, leucine, isoleucine, tyrosine and phenylalanine, whereas α-glucose and methylmalonic acid (MMA) were elevated in the term group. However, no significant metabolic changes in preterm and term infants were observed at week 2. Changes in stool metabolites were more pronounced at month 6 with the preterm group showing higher levels of faecal succinate, citrate and trimethylamine-N-oxide (TMAO), whereas the term infants showed higher levels of faecal β-hydroxybutyric acid (BHBA), fucose and pyruvatoxime. Additionally, the latter consistently showed elevated levels of BHBA and fucose up to month 12, whereas the preterm infants exhibited higher levels of faecal tyrosine and phenylalanine at month 12 as well as week 1.

**Table 3:** List of 1H NMR-derived metabolites that differ significantly between preterm and term infants at different time-points.

Time-point	Metabolites		Chemical shifts, ppm (multiplicity)^a^	References
	Relatively higher in preterm	Relatively higher in term		
Meconium Preterm (n = 15) versus Term(n = 19); R2X = 0.621; Q2Y = 0.179	Glycerol		3.5698(m), 3.6625(m), 3.7978(tt)	HMDB
Week 1 Preterm (n = 14) versus Term(n = 15); R2X = 0.39; Q2Y = 0.487		a-glucose	5.24(d), 3.56(dd), 3.70(t), 3.40(t), 3.83(m), 3.72(dd), 3.85(m)	HMDB (Merrifield et al.^70^)
		MMA	1.2362(d), 3.3074(q)	
	Valine		0.9936 (d), 1.0128 (d)	HMDB
	Leucine		0.9543 (m)	
	Isoleucine		0.9945(t), 1.012(d)	
	Tyrosine		7.2004 (d), 6.9097 (d)	
	Phenylalanine		7.335 (m), 7.3863 (m) ,7.4347 (m)	
Week 2 Preterm (n = 11) versus Term(n = 15); R2X = 0.228; Q2Y = 0.143	N.d	N.d		
Month 6 Preterm (n = 10) versus Term(n = 17); R2X = 0.7; Q2Y = 0.66	Succinate		2.4103 (s)	HMDB
	TMAO		3.3645 (s)	(Merrifield et al.^70^)
	Citrate		2.5488 (d), 2.6988 (d)	
		BHBA	1.2117 (d)	HMDB
		Fucose	1.2525 (d)	(Gomez-Gallego et al.^71^; Sundekilde et al.^72^)
		Pyruvatoxime	2.0642 (s)	
Month 12 Preterm (n = 8) versus Term(n = 17); R2X = 0.33; Q2Y = 0.216		Taurine	3.2572 (t), 3.4584 (t)	HMDB
		BHBA	1.2117 (d)	(Merrifield et al.^70^)
		Fucose	1.2525 (d)	
	Tyrosine		7.2004 (d), 6.9097 (d)	HMDB
	Phenylalanine		7.335 (m), 7.3863 (m) ,7.4347 (m)	

N.d = not detected. BHBA = β-hydroxybutyric acid. HMDB = Human Metabolome Database. MMA = Methylmalonic acid. TMAO = Trimethylamine N-oxide.

^a^Key: s = singlet, d = doublet, t = triplet, q = quartet, m = multiplet, dd = doublet of doublets , tt = triplet of triplets.

### Association between differentially expressed OTUs and significant metabolites

**Figure 4:**
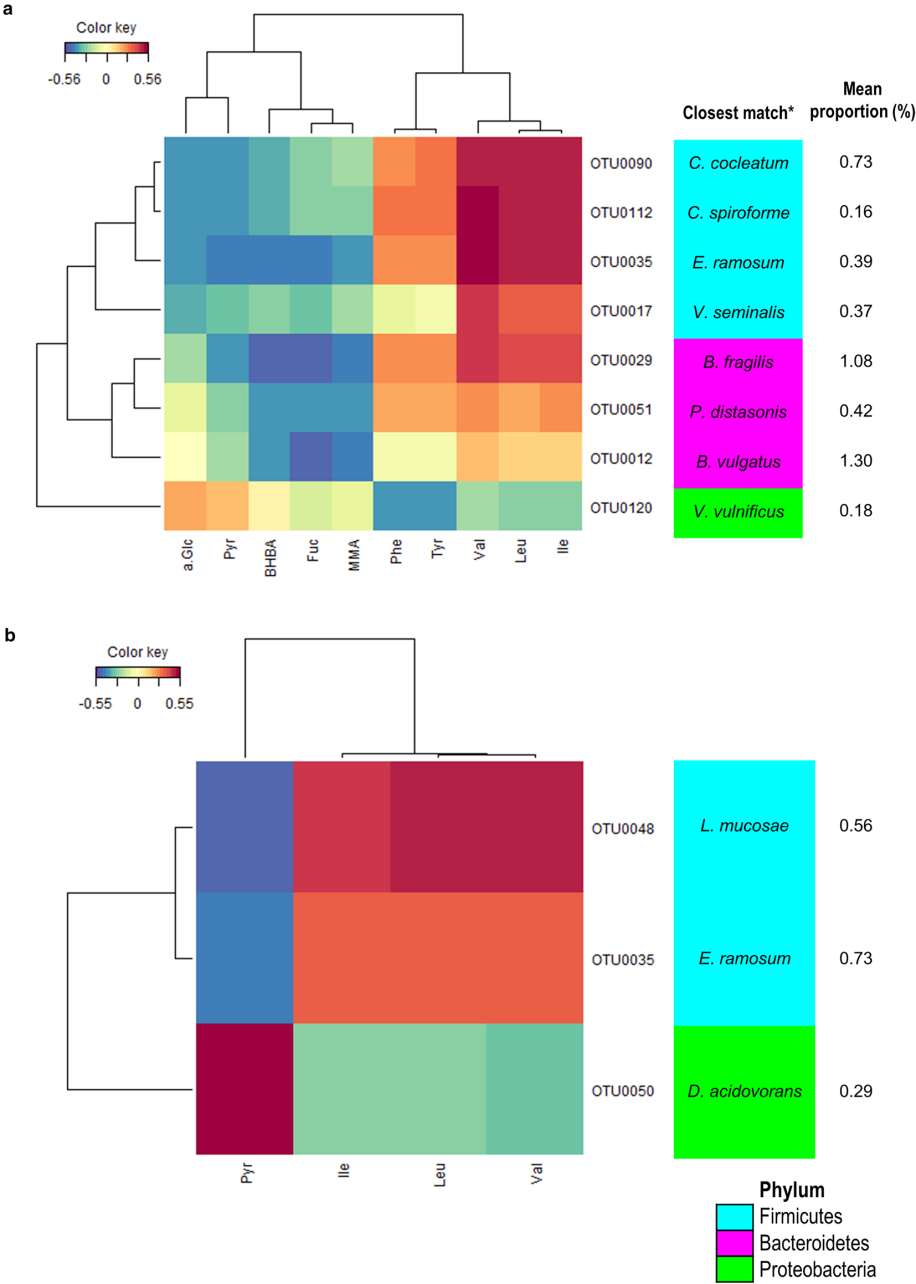
Spares partial least squared correlations (sPLS) between differentially expressed operational taxonomic units (OTUs) and identified significant metabolites in (**a**) preterm and (**b**) term groups. sPLS in regression mode (predict Y from X) to model a causal relationship between the OTUs and metabolites. Columns correspond to faecal metabolites; rows correspond to bacterial taxa. Red and blue denote positive and negative association, respectively. Percentage of mean proportion and bacterial phyla are summarised by the colour code on the right. Only the strongest pairwise associations were displayed, with a cut-off threshold of 0.4 (positive and negative). Heatmap plot was generated using R mixOmics package (version 3.5.0, https://CRAN.R-project.org/package=mixOmics). Key: a.Glu, α-glucose; BHBA, β-hydroxybutyric acid; Fuc, fucose; Ile, isoleucine; Leu, leucine; MMA, methylmalonic acid; Pyr, pyruvatoxime; Val, valine.

The relationship between differentially expressed OTUs and faecal metabolites were assessed using sparse partial least square model (sPLS-DA) (Fig. 4). In preterm, three *Clostridium*-related OTUs (OTU0035, OTU0090 and OTU0112) were positively correlated to the branched-chain amino acids (BCAAs): valine, leucine and isoleucine. In addition, OTU0035 (*Erysipelatoclostridium ramosum*) was negatively correlated to BHBA, fucose and pyruvatoxime while OTU0090 (*Clostridium cocleatum*) and OTU0112 (*Clostridium spiroforme*) were negatively correlated to pyruvatoxime and α-glucose. *Veillonella seminalis* (OTU0017) also showed correlations with the three BCAAs. *Bacteroides fragilis* (OTU0029) which was differentially expressed in term group at week 1 and week 2 and subsequently in preterm group at month 12, showed moderate to strong negative correlations to BHBA, fucose, MMA and pyruvatoxime. In term group, *Lactobacillus mucosae* (OTU0048) showed moderate to strong positive correlations with the three BCAAs and strong negative correlations with pyruvatoxime. OTU0050 which belongs to the genus *Delftia*, showed distinct clustering with negative correlations with the BCAAs and positive correlation with pyruvatoxime. OTU0050 was consistently significantly expressed in preterm group at week 1, month 6 and month 12, while term group, conversely, was enriched with the same OTU at week 2. Among the 10 OTUs with significant correlations, 50% of the taxa belonged to phylum Firmicutes while 20% of the taxa belonged to Proteobacteria. Importantly, the low abundant bacterial taxa present at relative abundance of less than 1% accounted for 65 total affecter correlations.

### Correlations between omics data with demographic and clinical parameters

Distance based linear modelling was carried out to identify the demographic and clinical predictors (Supplementary Data 1) for the differentially expressed 16S rRNA metagenomic and NMR metabolomic profiles. We modelled the faecal microbial and metabolic composition by splitting the data into each respective time point. Birth weight was consistently selected as the best explanatory variable for the total variation in the metabolic profiles all infants at birth and at month 12 (Table 4). On the other hand, gestational age was selected to explain the elevated 16S rRNA metagenomics profile for all infants at month 12. When the data was analysed by considering clinical parameters, the step-wise selection algorithm selected “PICC line insertion” and “isolation of bacteria resistant to third generation cephalosporins” as the best explanatory parameters for the faecal metabolic composition of the preterm group at month 6 and month 12 respectively.

**Table 4:** Cross-modelling of the stool microbial and metabolites profiles using a stepwise selection procedure.

Collection time	Variable	AICc	SS (trace)	Pseudo-F	*P*	Prop	Cumul	Res.df
**(A) Modelling of the differentially expressed gut microbial pattern of all infants using infant demographics as the predictor**								
Month 12	( +)Gestational age	172.85	5188.1	4.2861	0.001	0.16305	0.16305	22
**(B) Modelling of the elevated stool metabolites pattern of all infants using infant demographics as the predictor**								
Meconium	( +)Birth weight	− 186.74	0.041532	9.2098	0.003	0.21819	0.21819	33
Month 12	( +)Birth weight	− 146.81	0.0090759	4.565	0.019	0.17184	0.17184	22
**(C) Modelling of the elevated stool metabolites pattern of preterm infants using patient clinical information as the predictor**								
Month 6	( +)PICC line insertion (Yes/ No)	− 51.058	0.011965	3.7559	0.015	0.25031	0.53349	7
Month 12	( +)Isolation of bacteria resistant to 3^rd^ generation cephalosporins	− 41.009	0.0073412	4.9916	0.044	0.49958	0.49958	5

Prop. = explanatory proportion, Cumul. = cumulative explanatory proportion, Res.df = residual degree of freedom, ( +) = inclusion, PICC = Peripherally inserted central catheter. Only elements with significant effect are included.

## Discussion

In comparison to preterm infants, full term infants have completed in utero microbial and organ development. The relatively more matured organs provided distinct ecological landscape for the colonisation and establishment of the gut microbiota^21^. However, we showed that the difference in alpha diversities between term and preterm infants is generally unremarkable. This lack of difference in bacterial alpha diversities was also reported previously between moderate-late premature infants to term infants^9^. Nonetheless, significant differences in phylogenetic diversity between groups were observed at meconium and month 6, but not month 12, suggesting that the microbiome diversity of preterm may move towards similar level as term over time.

High Firmicutes:Bacteriodetes ratio of the gut microbes has been previously associated with a “healthier” gut. However, recent publications suggested that such link is over-simplified^17^. Owing to the diverse functional capability of each phylum, variation in “beneficial gut microbes” might not induce uniform phylum-wide metabolic responses. A change of associated external environment such as diet, infancy infections, living conditions are known to impact the composition of infant gut microbiome^22^. Although similar developmental patterns with respect to age and gut maturation were observed in various studies, the gut microbiota of healthy infants in a global comparison study exhibited different phylotypes. As reported by Kuang et al., majority of the gut microbiota of infants from western countries belonged to “A-type” (abundant in Actinobacteria), while most infants from Bangladesh were “F-type” (abundant in Firmicutes). In the same study, all healthy Chinese infants (age < 3 months) from China were “P-type” (abundant in Proteobacteria), with *Escherichia/ Shigella* and *Klebsiella* being the dominant genera^15^. Consistently, our data demonstrates that gut microbiota of all infants were dominated by Proteobacteria which gradually decreased thereafter, in line with the previous findings in which the abundance of Proteobacteria (mainly *Enterobacteriaceae*) gradually decreased from birth up to 24 months of life^23^. It was reported that geographical location exerted a stronger influence to the compositional differences in gut microbiome than other factors^24^. However, most major studies of early-life gut microbiome focused more on western cohorts^6,7^ and may not adequately represent the gut microbiota of the South East Asians which are ethnically diversified. Hence regional variations should be considered during data interpretation for the association of gut microbiota and infant health.

Throughout the years, human gut microbiome has been explored to provide better understanding of health and diseases. However, the accuracy of data reporting the abundance of bacterial gut microbiota is largely dependent on the primers and selected hypervariable regions^25^. Among the primers used in neonatal faecal 16S rRNA studies, the primers developed by Klindworth et al.^26^ was found to be able to amplify *Bifidobacterium* spp. compared to V4–V5 primers when the species is present in low abundance (< 0.05%)^27^. In a meta-analysis of full-term intestinal microbiota, 1035 publicly available datasets from 15 studies involving primer regions of V1–V2, V3, V4, V3–V4, and V4–V5 were assessed by using the web-tool TestPrime 1.0^28^. In general, all the primers studied produced comparable results. More specifically, the primers developed by Klindworth et al.^26^ showed an in silico efficiency of > 95% in amplifying the targeted *Bifidobacterium*, the important commensals of the infant gut microbiome^28^. In addition, Mazzola et al.^29^ have also reported a comparable performance of V3–V4 sequencing and qPCR in determining the abundance of *Bifidobacterium*. The bacterial membership in the stool collected in week 1 and week 2 displayed relatively higher consistency across individuals as compared to the meconium. The homogenisation effect observed might be a natural response of the gut microbiome towards the changes from womb to birth in which a smaller subset of available microbial populations was preferentially selected. It is notable that the divergence in preterm and term microbiomes was strongest at month 12. Previous study observed that the difference in abundance of *Klebsiella* spp. could last for more than 4 months, long after the initial neonatal period and it was correlated with later development of paediatric allergy^30^. Differentially abundance of potentially pathogenic *Klebsiella* OTUs observed among the term infants and their roles in the host immunological response and metabolism remained to be elucidated. The dichotomous development of the microbiome may be due to the available of the different species pools during birth (for e.g. natural birth vs. caesarean). The variation in physical development further resulted in the shift in gut microbial composition. These observations were consistent with the ecological processes such as “historical contingency” and “niche specialisation” ^31,32^.

In comparison to gut microbial composition, the metabolic profiles were relatively less sensitive to the infant development in terms of sampling time points. Temporal stability of the gut metabolome can be related to the concept of high redundancy of metabolic pathways across different microbial species^33,34^. Our finding is also consistent with previous literature suggesting that human metabolic profiles are more conserved than gut microbiome^34^. Elevated serum MMA and stool glucose have been associated with vitamin B12^35^ and carbohydrate malabsorption^36^ in children respectively. In this study, MMA and α-glucose were found to be significantly increased in week 1 stools of the term group. The sPLS-DA analysis showed that the elevation of stool MMA is negatively correlated with a *Parabacteroides* OTU (OTU0051) and two *Bacteroides* OTUs (OTU0012 and OTU0029). These four OTUs were differentially abundant in the stool samples of term group at week 1 and week 2, and only detected in preterm group at month 12. On the other hand, *Prevotella* OTUs were exclusively differentially expressed in preterm group at week 1, week 2 and month 6. The abundances of *Ruminococcaceae, Bacteroides,* and *Prevotella* had been proposed as enterotype identifiers which explained the most taxonomic variation across individuals^37^. Shift in *Prevotella-Bacteroides* microbial taxa has not been previously reported in infants.

The functional output of the gut microbiota, including amino acids and short-chain fatty acids (SCFAs) are thought to be important modulators underlying the pathogenesis of metabolic diseases such as obesity^38^, insulin resistance and type 2 diabetes mellitus^39^. Metabolites identified in the preterm group at week 1 include BCAAs such as valine, leucine and isoleucine, and aromatic amino acids including tyrosine and phenylalanine. High concentrations of neonatal faecal excretion of BCAAs and other amino acids metabolism derived metabolites indicate reduced protein absorption or excess protein intake^40^. Protein catabolism and absorption generally take place in the small intestine. During this process, amino acids that are not re-absorbed will be transferred to colon. We detected a positive correlation between BCAAs with three OTUs of *Erysipelatoclostridium* (former *Clostridium* spp.) and *Lactobacillus* (OTU0048). The former has been reported to be prevalent among the type 2 diabetes mellitus patients^41^ while *Clostridium ramosum* was detected in high-fat fed mice^42^. It was postulated that amino acids can serve as precursors for the synthesis of SCFAs by intestinal microbes to deliver additional energy to the host^38,43^. Thus, the elevated *Erysipelatoclostridium* (*Clostridium*) OTUs in preterm infants may play an important role in the energy metabolism that fuel rapid physical development. Succinate is a key intermediate in the tricarboxylic acid (TCA) cycle in host metabolism. The major producers of succinate in the mammalian gut are *Prevotella* spp.^44^. Although significantly higher abundance of *Prevotella* spp. in preterm as compared to term infants was detected, high succinate concentration was only observed in the stool samples collected at month 6. The lack of correlation might be due to the fact that succinate is only transiently available as it is rapidly converted as an intermediate in the production of propionate by the succinate-utilising bacteria^45^. Further, the high expression of succinate may also due to the extended administration of antibiotic among the preterm infants^46,47^. Another TCA cycle intermediate identified in preterm at month 6 is citrate, possibly indicating incomplete fermentation of complex polysaccharides in the gut of preterm. Additionally, the elevated TMAO identified in the preterm group at month 6 might be a response of weaning as the metabolite has been associated with animal proteins such as eggs, red meat and dairy^48^. TMAO is commonly identified in urine, but it could also be eliminated through faeces, although the levels of TMAO in faeces have not been carefully quantified^48^. In comparison with adults, children, especially infants have lower expression of TMAO precursors, thus it is less metabolised but the gut microbes^49^.

At 6 months of life, increased milk metabolites such as fucose and BHBA, as well as energy–related intermediate, pyruvatoxime, were observed in the term infants. Emerging evidence suggests the presence of fucose in mammalian gut could improve host health by supporting the competitive growth of beneficial members of the gut community^50^ and interact directly with host immune cells^51^. Studies have demonstrated the ability of *Bacteroides fragilis* to incorporate fucose into their own glycans for the production of fucosylated glycoproteins, which is necessary for its fitness to colonise the mammalian intestine^52–54^. Our finding supports this hypothesis in which *B. fragilis* was strongly and negatively associated with fucose. Fletcher et al*.*^52^ suggested that this general O-glycosylation system is conserved among the intestinal *Bacteroides* species. However, contrasting observations were noted in another study, in which *Bacteroides thetaiotaomicron* produces multiple fucosidases that cleave fucose from host glycans and contributes to the enterohaemorrhagic *E. coli* (EHEC) virulence^55^. This suggests that the beneficial or detrimental role of fucose in human gut is determined by the microbes which are responsible for its uptake. Besides being identified abundantly in milk, BHBA is long viewed as a ketone body that serves as a circulating energy source by the brain when blood glucose is low in times of fasting or prolonged exercise^56^. In this study, majority of the taxa with significant correlations in preterm group belonged to phylum Firmicutes and Bacteroidetes. Proteobacteria, despite being the most dominant phyla, might be transiently available in the neonatal gut^23^. Thus, when comparison was made, taxa that distinguish between term and preterm group were Firmicutes and Bacteroidetes.

One of the caveats of this study is the lack of detailed dietary data as the duration of breastfeeding, introduction to solid foods and cessation of milk feeding may influence the gut microbiota and subsequently the metabolites levels in the stool^57^. Nevertheless, feeding regimen of the preterm neonates during NICU admission was taken into account but none explained their stool microbial and metabolites patterns. Based on the DISTLM analysis of the overall elevated metabolites profile, birth weight was found to be the best explanatory variable at birth and at month 12 of life. In comparison, when the model was constructed by selecting the bacterial taxa that differed significantly in all infants, gestational age was associated with the gut microbial profiles at month 12. This is congruent with findings from one of the longest longitudinal preterm neonatal gut microbiota study in which gestational age continues to impact the microbial composition up to four years of age^58^. Similarly, La Rosa et al.^11^ also reported that the patterned progression of the gut bacterial community of preterm infants is most strongly influenced by gestational age at birth, than identifiable exogenous factors such as mode of delivery, antibiotic administration, or feeds^11^. When the DISTLM analysis was repeated on the differentially expressed metabolites of preterm infants with clinical variables during NICU admission, “PICC line insertion” and “isolation of bacteria resistant to third generation cephalosporins” were selected as the best explanatory variable at month 6 and month 12 respectively. Total parenteral nutrition (TPN) can be administered through umbilical catheters or PICCs, the latter should be used preferentially when prolonged PN is anticipated to prevent fewer insertion attempts and extravasation injuries to the neonates^59^. Data regarding changes in the microbiome and metabolome of humans receiving PICC insertion have been limited, but longitudinal alterations in the gut microbial colonisation patterns of infants who received TPN has been reported^60^. Nonetheless, blood microbiome signatures in neonatal PICCs associated with bloodstream infections were detected^61^. Although the aetiology for these observations on the neonatal microbiome is not clearly understood, we ascertain that PICC line insertion may exert a prolonged effect on the neonatal gut metabolism.

The metabolomic profiles showed that preterm infants harboured significantly more carbohydrate metabolism intermediates (citrate, succinate), BCAAs (leucine, isoleucine, valine) and aromatic amino acids (tyrosine, phenylalanine) in the stools through the first year of life as opposed to the term infants. Such differences might imply impaired sugar metabolism among the preterm infants. The metabolism of sugar is an important pathway for energy harvesting and essential of physical and mental growth in children^62^. In order to generate greater understanding in disease pathogenesis, there is a pressing need to extend the temporal coverage of infant gut microbial research. Despite of our modest sample size, this is the first targeted report on Malaysian preterm infant gut microbiome. Our study contributed to the understanding of the ethnically diverse South East Asian gut microbiota. In conclusion, we suggest that the early-life characteristics and environmental perturbations may exert a sustained effect of at least one year of life in the gut microbial composition and metabolism of the preterm neonates who were admitted to the NICU. Thus, our study highlighted the opportunities for large-scale studies to further investigate the association between early-life factors and a sustained microbiota imprint leading to establishment on methods of therapeutic intervention which could potentially lower the risk of developing metabolic dysfunction in later life.

## Methods

### Sample collection

Written informed consent was obtained from the infants’ parents and investigations were conducted according to the principles approved by the University of Malaya Research Ethics Committee (UMREC) with ethical approval number 201310–0267. The study was performed in accordance with the UMREC guidelines. Preterm infants were recruited among patients admitted to the neonatal intensive care unit (NICU) at University Malaya Medical Centre (Kuala Lumpur, Malaysia) from June 2014 to December 2014. Enrolled preterm infants were born at a gestational age (GA) of < 37 weeks. Clinical data and stool samples from diapers of preterm neonates were collected. Term neonates at a GA > 37 weeks and with no congenital malformations and metabolic diseases were recruited in this study as a comparative group. Stool samples were collected across five time points: at birth, week 1, week 2, month 6, and month 12, and same samples were subjected to both 16S rRNA sequencing and metabolomic profiling. Samples were immediately stored at − 20 °C and then transferred to − 80 °C no more than 3 days post-collection.

### DNA extraction, 16S rRNA sequencing and data processing

Nucleic acid extraction of stool was carried out on 220 mg of faecal sample using the QIAamp DNA Stool Mini Kit (Qiagen, Hilden, Germany) in accordance with the manufacturer’s instructions. PCR amplification of the 16S rRNA V3 and V4 regions was performed using primers V3-V4F (5′- TCG TCG GCA GCG TCA GAT GTG TAT AAG AGA CAG CCT ACG GGN GGC WGC AG -3′) and V3-V4R (5′- GTC TCG TGG GCT CGG AGA TGT GTA TAA GAG ACA GGA CTA CHV GGG TAT CTA ATC C -3′)^26^. The set of primers were carefully selected to minimise bias due to the mismatch of primers to amplify early-life microbial 16S rRNA genes (Supplementary Data 2). The reaction was performed with 1 µM each primer, 2×KAPA HiFi HotStart Ready Mix, 5 ng/µL of gDNA in 10 mM Tris pH 8.5 made up to 25 µL of reaction mixture. The thermoprofile used was as followed: initial denaturation for 3 min at 95 °C followed by 25 cycles of 95 °C for 30 s, 55 °C for 30 s and 72 °C for 30 s with a final extension at 72 °C for 5 min and hold at 4 °C. The PCR amplicons were purified by using Agencourt AMPure XP beads (Beckman Coulter) and the resultant DNA was quantified using a Qubit 3.0 Fluorometer (Life Technologies, Carlsbad, CA, United States) and Illumina sequencing adapters were attached to each sample using Nextera XT Index Kit. Amplicon fragment size was checked with a Bioanalyzer DNA 1000 chip and the DNA library qualities were evaluated with using the library quantification kit (KapaBiosystems) on an M×3000P qPCR system (Agilent Technologies). Following an initial library quantification using the MiSeq V2 reagent kit (Illumina) on the MiSeq system, the libraries were normalised and pooled at equimolar concentration with 15% PhiX spike-in. The final library was then subjected to paired-end 2 × 250 bp sequencing on a HiSeq 2500 platform using HiSeq rapid SBS kit v2 (Illumina).

Raw reads generated from Illumina paired-end sequencing were imported into Mothur software (version 1.39)^63^ for quality filtering and processing, according to the MiSeq SOP (https://mothur.org/wiki/miseq_sop/). Briefly, 99,122,189 paired-end sequences were joined into contigs. Contigs with ambiguous bases, homopolymer > 6 bp and sequence length below/ exceed the cut-off (< 460 bp and > 466 bp) were trimmed. Approximately 40% of the sequences were removed based on the filter and a total of 59,308,069 sequences were used for subsequent procedures. Chimeric sequences detected using Uchime command were removed before clustering the sequences into operational taxonomic units (OTUs). The taxonomic assignment was carried out in reference to SILVA database. Sequences affiliated with chloroplast, mitochondria, unknown, archaea, and eukaryote linages were further removed. Sequence for each OTU was blasted using NCBI database to identify the microorganisms at species level. The data was rarefied to equal depth for ease of comparison. Alpha diversity metrics including Shannon’s, Inverse Simpson’s, Pielou’s evenness, Chao1 and Faith’s phylogenetic diversity indices were calculated and projected in box plots using R microbiomeSeq package (https://github.com/umerijaz/microbiomeSeq). In additional, bar charts were constructed using phyloseq package in R^64^ to display the proportional differences in genus and phylum across groups. The beta-diversity was evaluated using statistical ordinations: Partial least squares discriminant analysis (PLS-DA) and Permutational Multivariate Analysis of Variance (PERMANOVA) from a Bray–Curtis distance matrix of normalised OTU counts. PLS-DA models were built by using the R mixOmics package^65^. PERMANOVA testing the inter-subject significance with time points was performed by using PRIMER + PERMANOVA software (version 7, PRIMER-E Ltd., Ivybridge, UK). Further, taxa showing significant differences in abundance between term and preterm infants at the respective time points were identified using negative log binomial model implemented in DESeq2 R package^66^. Correction of multiple corrections was conducted using Benjamini-Hochberg (BH) procedure implemented in DESeq2. Differentially expressed phylum genus and OTUs were selected by using BH adjusted P-value cut off of 0.01.

### Metabolomic analysis through nuclear magnetic resonance (NMR)

The faecal metabolite extraction was adapted from faecal metabolite extraction protocol as previously described by Yap et al.^67^. For each sample, a total of 0.05 g of faecal matter was homogenised with 1 mL of phosphate buffer (90% D2O,1 mM 3-trimethylsilyl-1-[2,2,3,3-2H4] propionate (TSP) and 3 mM sodium azide; pH 7.4). The homogenates were sonicated for 30 min in room temperature using a water bath sonicator followed by centrifugation at 13,000 rpm for 10 min. A total of 600 µL of supernatant was transferred to a 5 mm (outer diameter) NMR tube (Norell, USA) for NMR analysis.

A standard 1-dimensional (1D) 1H-NMR spectrum was acquired for each sample with a pulse [recycle delay (RD)–90°–t1–90°–tm–90°–acquire free induction decay (FID)] on a Bruker 600 MHz spectrometer (Bruker Biospin, Fallenden, Switzerland) with a 5 mm BB(F)O broadband probe operating at 600.13 MHz (ambient probe temperature 27 °C). The spectra were acquired according to parameters used previously^68^. The field frequency was locked on D2O solvent and water peak suppression was performed by gradient water pre-saturation during RD of 4 s and a mixing time (tm) of 0.01 s. The 90° pulse length was adjusted to ~ 10 μs and an acquisition time of 2.65 s was used.

Phasing and baseline correction of the 1H-NMR spectra were performed manually using Bruker TopSpin (version 4.0.5, Bruker Biospin, Fallenden, Switzerland). TSP was added in the samples as an internal reference peak (0.0 ppm) and the phosphate buffer (pH 7.4) was added to the samples to minimise peak shift due to residual pH changes. All spectra were referenced to the TSP resonance at δ 0.00. The spectra were digitised into 10 k data points using an in-house developed MATLAB (version R2014a, Natwick, USA) script^67^. The regions containing water resonances (δ 4.5–6.5) were removed from each spectrum to eliminate distortion effects of water peak on the baseline. Additionally, the regions, δ 0.0–0.5 and δ 9.2–10.0 in the stool water spectra which contain only noise were removed. Normalisation to the total sum of residual spectrum and data scaling to unit variance were also carried out prior to pattern recognition analysis.

The covariance plot was used to aid interpretation of the significance of each metabolite from the permutation tests. The significance and validity of statistical differences were calculated using permutation test (number of permutations = 1000). The colours projected onto the spectrum indicate significance of the metabolites with the blue indicating no significance difference at *P* ≥ 0.05 confidence levels. To minimise false positive results, a conservative cut-off of *P* < 0.01 (red) were used to select differentially abundant metabolites. All NMR metabolites were validated by 2D-NMR spectroscopy in accordance with in-house standards and database (O. Cloarec, Imperial College London).

### Integrated analysis of microbiome and metabolomics datasets

The relative abundance of the dominant bacterial taxa from 16S rRNA gene sequencing and the concentration of selected NMR metabolites were integrated using sparse partial least squares regression (sPLS) implemented under R mixOmics package^65^. Successive samples collected from the same individual were treated as independent sample considering that the biological variations between subjects are independent where different conditions are applied on the same subject during repeated measurements^69^. Heat map chart was constructed to observe the association of metabolomics and metagenomics features.

### Distance-based linear modelling (DISTLM)

DISTLM was performed to identify the association between the clinical and demographic parameters with microbial assemblage pattern and changes in metabolic profiles^68^. In brief, the clinical and demographic parameters were selected and fitted to the overall changes in gut microbial composition and metabolic profiles using stepwise regression under the second-order bias-corrected Akaike Information Criterion (AIC).

## Supplementary information

Supplementary Information 1.

## Data Availability

The 16S rRNA dataset generated and analysed during the current study is available in the NCBI Sequence Read Archive (PRJNA578822; available at https://www.ncbi.nlm.nih.gov/sra/PRJNA578822) and metabolomics data is available from the corresponding author on reasonable request.

## References

[1] Morrison KM (2016). Cardiometabolic health in adults born premature with extremely low birth weight. Pediatrics.

[2] Crump C, Winkleby MA, Sundquist K, Sundquist J (2011). Risk of diabetes among young adults born preterm in Sweden. Diabetes Care.

[3] Naumburg E, Soderstrom L (2019). Increased risk of pulmonary hypertension following premature birth. BMC Pediatr..

[4] Crump C, Winkleby MA, Sundquist K, Sundquist J (2011). Risk of hypertension among young adults who were born preterm: a Swedish national study of 636,000 births. Am. J. Epidemiol..

[5] Mathai S (2013). Increased adiposity in adults born preterm and their children. PLoS ONE.

[6] Stewart CJ (2018). Temporal development of the gut microbiome in early childhood from the TEDDY study. Nature.

[7] Backhed F (2015). Dynamics and stabilization of the human gut microbiome during the first year of life. Cell Host Microbe.

[8] Korpela K (2018). Intestinal microbiota development and gestational age in preterm neonates. Sci. Rep..

[9] Chernikova DA (2018). The premature infant gut microbiome during the first 6 weeks of life differs based on gestational maturity at birth. Pediatr. Res..

[10] Arboleya S (2012). Establishment and development of intestinal microbiota in preterm neonates. FEMS Microbiol. Ecol..

[11] La Rosa PS (2014). Patterned progression of bacterial populations in the premature infant gut. Proc. Natl. Acad. Sci. U.S.A..

[12] Mshvildadze M (2010). Intestinal microbial ecology in premature infants assessed with non-culture-based techniques. J. Pediatr..

[13] Gibson MK (2016). Developmental dynamics of the preterm infant gut microbiota and antibiotic resistome. Nat. Microbiol..

[14] Shi YC (2018). Initial meconium microbiome in Chinese neonates delivered naturally or by cesarean section. Sci. Rep..

[15] Kuang YS (2016). Composition of gut microbiota in infants in China and global comparison. Sci. Rep..

[16] Subramanian S (2014). Persistent gut microbiota immaturity in malnourished Bangladeshi children. Nature.

[17] Priya S, Blekhman R (2019). Population dynamics of the human gut microbiome: change is the only constant. Genome Biol..

[18] Tauchi H (2019). Gut microbiota development of preterm infants hospitalised in intensive care units. Benef. Microbes.

[19] Preliminary Report of National Obstetrics Registry, Jan 2013–Dec 2015. Malaysia National Obstetrics Registry. (2017).

[20] Yap PS (2016). Intestinal carriage of multidrug-resistant gram-negative bacteria in preterm-infants during hospitalization in neonatal intensive care unit (NICU). Pathog. Glob. Health.

[21] DiBartolomeo ME, Claud EC (2016). The developing microbiome of the preterm infant. Clin. Ther..

[22] Gschwendtner S (2019). Early life determinants induce sustainable changes in the gut microbiome of six-year-old children. Sci. Rep..

[23] Niu J (2020). Evolution of the gut microbiome in early childhood: A cross-sectional study of Chinese children. Front. Microbiol..

[24] He Y (2018). Regional variation limits applications of healthy gut microbiome reference ranges and disease models. Nat. Med..

[25] Brooks JP (2015). The truth about metagenomics: quantifying and counteracting bias in 16S rRNA studies. BMC Microbiol..

[26] Klindworth A (2013). Evaluation of general 16S ribosomal RNA gene PCR primers for classical and next-generation sequencing-based diversity studies. Nucleic Acids Res..

[27] Biol-Aquino MA, Perdiz CJ, Borlagdan M, Alcantara JD, Mallillin A (2019). Differences in the bacterial profiles of infant gut by birth process, milk diet, and choice of 16S rRNA gene target region. Hum. Microbiome J..

[28] Mancabelli L (2020). Multi-population cohort meta-analysis of human intestinal microbiota in early life reveals the existence of infant community state types (ICSTs). Comput. Struct. Biotechnol. J>.

[29] Mazzola G (2016). Early gut microbiota perturbations following intrapartum antibiotic prophylaxis to prevent group B *Streptococcal* Disease. PLoS ONE.

[30] Reyman M (2019). Impact of delivery mode-associated gut microbiota dynamics on health in the first year of life. Nat. Commun..

[31] Costello EK, Stagaman K, Dethlefsen L, Bohannan BJ, Relman DA (2012). The application of ecological theory toward an understanding of the human microbiome. Science.

[32] Vieira-Silva S (2016). Species-function relationships shape ecological properties of the human gut microbiome. Nat. Microbiol..

[33] Lozupone CA, Stombaugh JI, Gordon JI, Jansson JK, Knight R (2012). Diversity, stability and resilience of the human gut microbiota. Nature.

[34] Visconti A (2019). Interplay between the human gut microbiome and host metabolism. Nat. Commun..

[35] Sentongo TA, Azzam R, Charrow J (2009). Vitamin B12 status, methylmalonic acidemia, and bacterial overgrowth in short bowel syndrome. J. Pediatr. Gastroenterol. Nutr..

[36] Hammer HF (1990). Carbohydrate malabsorption. Its measurement and its contribution to diarrhea. J. Clin. Investig..

[37] Falony G (2016). Population-level analysis of gut microbiome variation. Science.

[38] Turnbaugh PJ (2006). An obesity-associated gut microbiome with increased capacity for energy harvest. Nature.

[39] Chen S (2019). Serum amino acid profiles and risk of type 2 diabetes among Japanese adults in the Hitachi Health Study. Sci. Rep..

[40] Bridgman SL (2017). Fecal short-chain fatty acid variations by breastfeeding status in infants at 4 months: Differences in relative versus absolute concentrations. Front. Nutr..

[41] Qin J (2012). A metagenome-wide association study of gut microbiota in type 2 diabetes. Nature.

[42] Woting A, Pfeiffer N, Loh G, Klaus S, Blaut M (2014). Clostridium* ramosum* promotes high-fat diet-induced obesity in gnotobiotic mouse models. mBio.

[43] Serena C (2018). Elevated circulating levels of succinate in human obesity are linked to specific gut microbiota. ISME J..

[44] Reichardt N (2014). Phylogenetic distribution of three pathways for propionate production within the human gut microbiota. ISME J..

[45] Louis P, Flint HJ (2017). Formation of propionate and butyrate by the human colonic microbiota. Environ. Microbiol..

[46] Tulstrup MV (2015). Antibiotic treatment affects intestinal permeability and gut microbial composition in wistar rats dependent on antibiotic class. PLoS ONE.

[47] Woodmansey EJ, McMurdo ME, Macfarlane GT, Macfarlane S (2004). Comparison of compositions and metabolic activities of fecal microbiotas in young adults and in antibiotic-treated and non-antibiotic-treated elderly subjects. Appl. Environ. Microbiol..

[48] Janeiro MH, Ramirez MJ, Milagro FI, Martinez JA, Solas M (2018). Implication of trimethylamine N-oxide (TMAO) in disease: Potential biomarker or new therapeutic target. Nutrients.

[49] Fennema D, Phillips IR, Shephard EA (2016). Trimethylamine and trimethylamine N-oxide, a flavin-containing monooxygenase 3 (FMO3)-mediated host-microbiome metabolic axis implicated in health and disease. Drug Metab. Dispos. Biol. Fate Chem..

[50] Pham TA (2014). Epithelial IL-22RA1-mediated fucosylation promotes intestinal colonization resistance to an opportunistic pathogen. Cell Host Microbe.

[51] Pickard JM, Chervonsky AV (2015). Intestinal fucose as a mediator of host-microbe symbiosis. J. Immunol..

[52] Fletcher CM, Coyne MJ, Villa OF, Chatzidaki-Livanis M, Comstock LE (2009). A general O-glycosylation system important to the physiology of a major human intestinal symbiont. Cell.

[53] Coyne MJ, Reinap B, Lee MM, Comstock LE (2005). Human symbionts use a host-like pathway for surface fucosylation. Science.

[54] Coyne MJ, Chatzidaki-Livanis M, Paoletti LC, Comstock LE (2008). Role of glycan synthesis in colonization of the mammalian gut by the bacterial symbiont Bacteroides fragilis. Proc. Natl. Acad. Sci. U.S.A..

[55] Pacheco AR (2012). Fucose sensing regulates bacterial intestinal colonization. Nature.

[56] Newman JC, Verdin E (2014). Ketone bodies as signaling metabolites. Trends Endocrinol. Metab.: TEM.

[57] Laursen MF, Bahl MI, Michaelsen KF, Licht TR (2017). First foods and gut microbes. Front. Microbiol..

[58] Fouhy F (2019). Perinatal factors affect the gut microbiota up to four years after birth. Nat. Commun..

[59] Bolisetty S (2020). Standardised neonatal parenteral nutrition formulations—Australasian neonatal parenteral nutrition consensus update 2017. BMC Pediatr..

[60] Dahlgren AF (2019). Longitudinal changes in the gut microbiome of infants on total parenteral nutrition. Pediatr. Res..

[61] Pammi M (2020). Microbiome signatures in neonatal central line associated bloodstream infections. PLoS ONE.

[62] Henderickx JGE, Zwittink RD, van Lingen RA, Knol J, Belzer C (2019). The preterm gut microbiota: An inconspicuous challenge in nutritional neonatal care. Front. Cell. Infect. Microbiol..

[63] Schloss PD (2009). Introducing Mothur: open-source, platform-independent, community-supported software for describing and comparing microbial communities. Appl. Environ. Microbiol..

[64] McMurdie PJ, Holmes S (2013). phyloseq: an R package for reproducible interactive analysis and graphics of microbiome census data. PLoS ONE.

[65] Rohart F, Gautier B, Singh A, Le Cao KA (2017). mixOmics: An R package for 'omics feature selection and multiple data integration. PLoS Comput. Biol..

[66] Love MI, Huber W, Anders S (2014). Moderated estimation of fold change and dispersion for RNA-seq data with DESeq2. Genome Biol..

[67] Yap IK (2008). Metabonomic and microbiological analysis of the dynamic effect of vancomycin-induced gut microbiota modification in the mouse. J. Proteome Res..

[68] Yap IK (2015). Acclimatisation-induced stress influenced host metabolic and gut microbial composition change. Mol. BioSyst..

[69] Liquet B, Le Cao KA, Hocini H, Thiebaut R (2012). A novel approach for biomarker selection and the integration of repeated measures experiments from two assays. BMC Bioinform..

[70] Merrifield, C. A. *et al.* Neonatal environment exerts a sustained influence on the development of the intestinal microbiota and metabolic phenotype. *ISME J.* **10**(1), 145–157. 10.1038/ismej.2015.90 (2016). 10.1038/ismej.2015.90 PMC4681865 26066712

[71] Gomez-Gallego, C. *et al.* Human breast milk NMR metabolomic profile across specific geographical locations and its association with the milk microbiota. *Nutrients**10*(10). 10.3390/nu10101355 (2018). 10.3390/nu10101355 PMC6213536 30248972

[72] Sundekilde, U. K., Larsen, L. B. & Bertram, H. C. (2013). NMR-based milk metabolomics. *Metabolites*, **3**(2), 204–222. 10.3390/metabo3020204 10.3390/metabo3020204 PMC3901264 24957988

