# Publisher Correction: Neonatal intensive care unit (NICU) exposures exert a sustained influence on the progression of gut microbiota and metabolome in the first year of life

**DOI:** 10.1038/s41598-021-88758-8

**Published:** 2021-04-21

**Authors:** Polly Soo Xi Yap, Chun Wie Chong, Azanna Ahmad Kamar, Ivan Kok Seng Yap, Yao Mun Choo, Nai Ming Lai, Cindy Shuan Ju Teh

**Affiliations:** 1 Department of Medical Microbiology, Faculty of Medicine, University of Malaya, 50603 Kuala Lumpur, Malaysia; 2 School of Pharmacy, Monash University Malaysia, 47500 Bandar Sunway, Selangor, Malaysia; 3 Neonatal Intensive Care Unit (NICU), Department of Paediatrics, Faculty of Medicine, University of Malaya, 50603 Kuala Lumpur, Malaysia; 4 Sarawak Research and Development Council, 11Th Floor LCDA Tower, The Isthmus, 93050 Kuching, Sarawak Malaysia; 5 School of Medicine, Faculty of Health and Medical Sciences, Taylor’s University, 47500 Subang Jaya, Selangor, Malaysia

Correction to:* Scientific Reports* 10.1038/s41598-020-80278-1, published online 14 January 2021

In the original version of this Article, Yao Mun Choo was incorrectly affiliated with ‘School of Pharmacy, Monash University Malaysia, 47500 Bandar Sunway, Selangor, Malaysia’. The correct affiliation is listed below.

Neonatal Intensive Care Unit (NICU), Department of Paediatrics, Faculty of Medicine, University of Malaya, 50603 Kuala Lumpur, Malaysia.

This error has now been corrected in the PDF and HTML versions of the Article.

