# Supplementary material for: Neonatal intensive care unit (NICU) exposures exert a sustained influence on the progression of gut microbiota and metabolome in the first year of life
Source: Sci Rep. 2021 Jan 14;11:1353. doi: 10.1038/s41598-020-80278-1 (PMC7809424; doi:10.1038/s41598-020-80278-1)
Supplement: Supplementary file 1 — Supplementary Information 1. [file 41598_2020_80278_MOESM1_ESM.docx]

**Neonatal intensive care unit (NICU) exposures exert a sustained influence on the progression of gut microbiota and metabolome in the first year of life**

**Author Correction:** In the original version of this Article, Yao Mun Choo was incorrectly affiliated with ‘School of Pharmacy, Monash University Malaysia, 47500 Bandar Sunway, Selangor, Malaysia’. The correct affiliation is listed below.

Neonatal Intensive Care Unit (NICU), Department of Paediatrics, Faculty of Medicine, University of Malaya, 50603 Kuala Lumpur, Malaysia.

**Revised affiliation implemented:**

Polly Soo Xi Yap^1,ꝉ^, Chun Wie Chong^2,ꝉ^, Azanna Ahmad Kamar^3^ , Ivan Kok Seng Yap^4^,

Yao Mun Choo^3^, Nai Ming Lai^5^, Cindy Shuan Ju Teh^1,*^

^1^Department of Medical Microbiology, Faculty of Medicine, University of Malaya, 50603 Kuala Lumpur, Malaysia.

^2^School of Pharmacy, Monash University Malaysia, 47500 Bandar Sunway, Selangor, Malaysia.

^3^Neonatal Intensive Care Unit (NICU), Department of Paediatrics, Faculty of Medicine, University of Malaya, 50603 Kuala Lumpur, Malaysia.

^4^Sarawak Research and Development Council, 11th Floor LCDA Tower, The Isthmus, 93050 Kuching, Sarawak.

^5^School of Medicine, Faculty of Health and Medical Sciences, Taylor’s University, 47500 Subang Jaya, Selangor, Malaysia.

*Correspondence:

Cindy Shuan Ju Teh

[cindysjteh@um.edu.my](mailto:cindysjteh@um.edu.my)

^ꝉ^These authors contributed equally to this work.
